# Structure-guided virtual screening reveals phytoconstituents as potent cathepsin B inhibitors: Implications for cancer, traumatic brain injury, and Alzheimer’s disease

**DOI:** 10.3389/fmolb.2025.1581711

**Published:** 2025-04-16

**Authors:** Nageeb Hassan, Mohammad Furkan, Mohd Shahnawaz Khan, Azna Zuberi, Moyad Shahwan, Anas Shamsi

**Affiliations:** ^1^ Department of Clinical Sciences, College of Pharmacy and Health Sciences, Ajman University, Ajman, United Arab Emirates; ^2^ Department of Biochemistry, Aligarh Muslim University, Aligarh, India; ^3^ Department of Biochemistry, King Saud University, Riyadh, Saudi Arabia; ^4^ Division of Reproductive Science in Medicine, Department of Obstetrics & Gynecology, Feinberg School of Medicine, Northwestern University, Chicago, IL, United States; ^5^ Center for Medical and Bio-Allied Health Sciences Research, Ajman University, Ajman, United Arab Emirates

**Keywords:** cathepsin B, cancer, traumatic brain injury, Alzheimer’s disease, drug discovery, virtual screening hERG blocker hepatotoxicity

## Abstract

Cathepsin B (CathB) is a lysosomal cysteine protease involved in various pathological and physiological processes and is becoming an attractive target for drug intervention in complex diseases like cancer, traumatic brain injury (TBI) and Alzheimer’s disease (AD). The aberrant expression of CathB drives tumor invasiveness and metastasis and exacerbates neurodegeneration and behavioral deficits in AD and TBI. However, current CathB inhibitors lack clinical translation due to poor selectivity, bioavailability, or toxicity, necessitating novel therapeutic candidates. To address this gap, an *in silico* screening was conducted through the structure-guided virtual screening with the IMPPAT 2 phytochemical library for potential CathB inhibitors. Using the control inhibitor CA-074Me as a benchmark, two phytoconstituents, Nicandrenone and Picrasidine M, emerged with superior binding affinities, ligand efficiency, and robust interactions with the active site residues of CathB. These molecules were further validated through molecular dynamics (MD) simulations, which supported their ability to bind stably to the CathB active pocket and thus likely hold their durable inhibitory activity. Remarkably, these phytoconstituents exhibited favorable pharmacokinetic and ADMET profiles, which validate their potential as lead compounds. The current study showed that these bioactive compounds could be developed as new CathB inhibitors, opening a new frontier for their use in the management of such diseases as cancer, TBI, and AD.

## 1 Introduction

Proteases constitute a diverse enzyme group which breaks protein peptide bonds to perform essential functions in biological processes, including protein turnover, cell signaling and inflammation, and blood coagulation (Jamal et al., 2024). Among these, cathepsin B (CathB), a lysosomal cysteine protease of the papain-like family, exhibits a dual role in both physiological and pathological states (Stoka et al., 2023). The enzyme CathB normally resides in lysosomes to perform protease and peptide breakdown activities (Aggarwal and Sloane, 2014). The deregulation of this protease has been strongly associated with cancer development and traumatic brain injury (TBI) along with Alzheimer’s disease (AD) as well as additional diseases (Wang et al., 2023). Due to its broad participation in pathological processes, it becomes an attractive candidate for therapeutic intervention (Hook et al., 2015). Research on CathB in cancer settings has intensified because this enzyme contributes to cancer cell movement as well as metastasis formation (Mijanović et al., 2019).

CathB has been reported to break down ECM components, which constitute a critical event in invasion and metastasis (Mijanović et al., 2019). Increased CathB expression and its secretion into the ECM enable the degradation of structural substrata and tumor cell invasion of surrounding tissues and the formation of metastases in distant organs (Kryczka et al., 2019). Additionally, CathB has been found to activate other proteases, such as the matrix metalloproteinases (MMPs), all of which play a role in the degradation of ECM and increased tumor-invasive properties (Fonović and Turk, 2014). The activity of CathB also affects the signaling pathways that promote oncogenesis independently from its protease function. The improper regulation of CathB leads to enhanced cellular growth alongside decreased cell death, together with new blood vessel formation, which are all cancer-related features (Gondi and Rao, 2013). The reduction of tumor burden and metastatic properties has been achieved through experimental models using small molecule inhibitors along with oligonucleotides or RNA to inhibit CathB activity (Petruzzella, 2024). Oncology drug development benefits from CathB as an important target because studies demonstrate its vital role in therapeutic progress.

Alzheimer’s disease (AD) is a chronic neurodegenerative disease that results in dementia, which is the gradual decline in memory and other aspects of the personality. One of the major characteristics of AD is the deposition of neurotoxic β-amyloid (Aβ) peptides in the brain and the development of amyloid plaques and subsequent neuronal injury. CathB is involved in the cleavage of Aβ precursor protein (APP) with the result that it produces Aβ peptides (Wang et al., 2012). CathB is involved in the processing of APP at certain sites to produce Aβ(1–40) and Aβ(1–42), which are the main components of amyloid plaques (Mueller-Steiner et al., 2006). However, the function of CathB in AD far exceeds the contribution of Aβ. It has been reported that CathB activity is higher in the AD-affected brain, and the degree of increase is proportional to the severity of the disease and the extent of cognitive impairment (Wu et al., 2023). Research using CathB inhibitors on AD animal models revealed lower Aβ concentrations with simultaneous improvements of synaptic function and cognitive performance (Hook et al., 2011). The observed research data supports CathB as a viable therapeutic target to control Aβ toxicity and protect neurons from decay in AD.

Another condition in which CathB is known to be significantly involved in TBI (Hook et al., 2020). It is well known that TBI is connected with neuroinflammation, neuronal damage and cognitive deficits, all of which are regulated by CathB (Luo et al., 2010). Increased CathB has been found in TBI subjects, and it degrades ECM, weakens the BBB, and triggers inflammation (Hook et al., 2020). The suppression of CathB in animal models of TBI has shown positive effects in cognitive and behavioral interventions, highlighting that the inhibition of the enzyme is a potential therapy (Hook et al., 2015). However, several issues should be addressed more carefully regarding CathB inhibitors as potential therapeutic agents: 1) The absence of selective CathB inhibitors; 2) The lack of understanding of the precise relationship between CathB activity and diseases; 3) The selectivity of CathB inhibitors for cancer cells and the difficulty of delivering these inhibitors to the tumor site (Zamyatnin et al., 2022; Kos et al., 2014). CathB is expressed in all tissues and implicated in critical physiological functions; however, it appears to have off-target effects and toxicity. Moreover, the enzyme’s structural versatility and active site promiscuity make it challenging to develop selective inhibitors. Existing inhibitors, while effective in preclinical models, often lack the selectivity and pharmacokinetic properties required for clinical use (Saroha et al., 2022). Therefore, there is a pressing need to identify novel molecules that exhibit improved efficacy, selectivity, and safety profiles.

Over time, computational approaches have emerged as viable solutions by presenting a cheaper and more efficient way of developing drugs (Vicidomini et al., 2024). Of these, virtual screening has been particularly proven to be a reliable method for determining the inhibitors of target enzymes (Alrouji et al., 2025). This technique involves the virtual screening of a large database of chemical compounds in order to select from the database the compounds with high binding probability to the active site of the target. As a tool that can be employed alongside molecular docking and molecular dynamics (MD), virtual screening permits the prediction of the target and potential inhibitors (Shamsi et al., 2024a). Most importantly, CathB’s virtual screening is highly effective in case of selecting lead compounds with high specificity and potency (Chitranshi et al., 2021). With the help of the structural data from crystallography and computational modeling, one can address CathB’s active site and design potent inhibitors (Jangra et al., 2024). Furthermore, MD simulations provide valuable insights about the stability and dynamics of the enzyme-inhibitor complexes and the enhancement of lead compounds.

It is widely accepted that natural products, especially phytochemicals, are potential sources of bioactive compounds with therapeutic properties (Narayanankutty et al., 2024). Phytochemicals have been superior to synthetic molecules as they possess structural diversities, possess inherent biological activity and are less toxic (Sun and Shahrajabian, 2023). A number of plant compounds have already been reported to possess protease inhibitory properties, and, therefore, they could be potential lead compounds for the modulation of CathB (Moon, 2024). In the present investigation, we intended to screen bioactive phytochemicals as CathB inhibitors using a structure-based computational strategy. For our study, we used the IMPPAT 2.0 database, containing 17,967 phytochemicals isolated from native Indian medicinal plants (Vivek-Ananth et al., 2023). This database serves as a rather specific focus for studying the possible application of different compounds of natural origin in therapy. Thus, the objectives of the present study were to virtually screen phytochemicals with high binding affinities, followed by molecular docking, pharmacokinetic profiling, and MD simulations to determine the stability of the phytochemicals with CathB’s active site. The combination of computational methods with natural product chemistry is a path to further enhancing the drug discovery process and identifying new therapeutic opportunities in the treatment of multifactorial diseases. The outcomes emphasize the therapeutic application of plant CathB inhibitors and provide direction for further experimental and clinical research.

## 2 Materials and methods

### 2.1 Receptor and grid preparation

To analyze the structural and functional properties of CathB, its three-dimensional (3D) structure was obtained from the Protein Data Bank (PDB ID: 1GMY) (Burley et al., 2022; Greenspan et al., 2001). This structure was selected due to its high resolution and relevance in CathB inhibitor studies. The structure was renumbered with PyMOL based on the UniProt entry (https://www.uniprot.org/uniprotkb/P07858). The optimized CathB structure was selected as the receptor for the molecular docking study. The structure was exported to MGL AutoDock Tools (Goodsell et al., 2021) in the PDB format to assign the right atom type. In order to provide comprehensive screening, InstaDock 1.2 was used to establish a docking grid around the whole protein structure (Mohammad et al., 2021). The grid box size was chosen to be 61 Å × 64 Å × 60 Å with a grid spacing of 19.881 Å, 37.74 Å, and 37.191 Å for the X, Y, and Z-axes, respectively. The docking was then performed using the default algorithm and scoring function of InstaDock by predicting the binding position and orientation of ligands.

### 2.2 Small molecular dataset

Twelve thousand phytochemical compounds were retrieved from the IMPPAT 2.0 database (https://cb.imsc.res.in/imppat/) as the dataset, which enriched the chemical structures and biological activities (Vivek-Ananth et al., 2023). The phytochemicals chosen for this virtual screening were from Indian medicinal plants and were primarily filtered based on their physicochemical properties following Lipinski’s rule of five. The ligands were analyzed and prepared using AutoDock tools; this involved correct assignment of atom types, preservation of stereochemistry, and optimization of the ionization states of the compounds. The prepared dataset was then imported into InstaDock for molecular docking-based virtual screening.

### 2.3 Molecular docking workflow

The virtual screening protocol applied the blind grid-based ligand docking with energy calculations on all the compounds of the IMPPAT 2 dataset (Shamsi et al., 2024b). The grid box used in InstaDock was large enough to allow for all the ligands to move and predict their potential binding site(s) on CathB. Binding affinity and ligand efficiency were determined for all the docked conformations. To analyze the three-dimensional binding prototype of the compounds with CathB, the best-scoring ligand poses based on binding energy and the docking scores were selected using InstaDock v1.2 (https://hassanlab.org/instadock). These protein-ligand interactions were then used to determine potential CathB binders.

### 2.4 Pharmacokinetic analysis

After the docking process, the selected compounds were subjected to pharmacokinetic and physicochemical property analysis using absorption, distribution, metabolism, excretion, and toxicity (ADMET) prediction tools. The Deep-PK (https://biosig.lab.uq.edu.au/deeppk) (Myung et al., 2024) tool was used to predict the ADMET properties of the compounds. Furthermore, the compounds were also filtered using the PAINS filtration to remove compounds with interference in the assays (Baell, 2016). Thus, only compounds with favorable pharmacokinetic and ADMET profiles and no PAINS patterns were considered for the subsequent simulation study. PAINS filtering was performed post-docking to avoid prematurely discarding promising compounds.

### 2.5 Molecular dynamics simulations

To analyze the stability and structural dynamics of CathB and its docked complexes, all-atom MD simulations were carried out. The analyses were performed with the GROMACS software package for 500 ns (Van Der Spoel et al., 2005). The CathB-ligand complexes were solvated in a cubic box containing SPC216 water molecules, where appropriate amounts of Na^+^ and Cl^−^ions were added to the system to achieve a physiological ionic strength of 0.1 M. The molecular mechanics parameterization scheme used in generating the topology files was the GROMOS 54A7 force field. The energy minimization was performed on the basis of the steepest-decent algorithm with the number of steps being 5000 for 1 nanosecond. This stabilization was done under constant temperature and volume (NVT) and constant temperature and pressure (NPT) with the simulation done for 2 ns at 300 K and 1 ATM, respectively. Hydrogen bonding was curbed by the SHAKE algorithm (Kräutler et al., 2001) All the production MD simulations were carried out for 500 ns, and the trajectory analysis of all the MD simulations were done with the help of GMX tools depending on various systematic parameters.

### 2.6 MM/PBSA calculations

The Molecular Mechanics/Poisson-Boltzmann Surface Area (MM/PBSA) method is commonly used to estimate the binding free energy between proteins and ligands (Genheden and Ryde, 2015). It combines molecular mechanics calculations with solvation energy approximations, to provide useful information about the stability of a molecule and its affinity towards other molecules. For this study, MM/PBSA analysis was used to measure the binding free energies for the CathB-ligand complexes. For accurate prediction of binding interactions, the last 10 ns of each of the MD simulation was taken from the steady state. The binding free energy components were computed using gmx_mmpbsa package which is based on the MM/PBSA approach depending on the following equation.

$$\Delta G_{\text{Binding}}=G_{\text{Complex}}-\left(G_{\text{Protein}}+G_{\text{Ligand}}\right)$$

where *G*_Complex_ signifies the total free energy of the binding complex, and *G*_Protein_ and *G*_Ligand_ are the measure of total free energies of CathB and the bound ligands, respectively.

## 3 Results and discussion

### 3.1 Molecular docking-based virtual screening

Molecular docking based virtual screening is another significant process in the drug discovery procedure that helps in avoiding problems of computational chemistry approaches by selecting potential molecules out of large data sets (Zhang et al., 2022). When targeting at CathB, 11,908 phytochemicals from the IMPPAT 2.0 database were screened according to their 3D conformations. For this purpose, the compound Ca- 074Me was used as a control molecule for the comparison of the docking results. The docking study was conducted using InstaDock and out of all the molecules, the top 10 molecules with the binding free energies between – 9.5 and – 10.3 kcal/mol were considered for the study. The binding affinities and ligand efficiencies of the mentioned hits and the control molecule are listed in detail in Table 1. These results demonstrated that all these phytochemicals had high binding affinity towards CathB. Notably, all identified hits exhibited superior binding affinities compared to the control molecule CA-074Me, which showed a binding affinity of −6.5 kcal/mol (Table 1). Ca-074Me has a documented IC_50_ of 2.24 nM and is selective for CathB (Steverding, 2011). Given that higher docking scores correlate with more substantial binding potential and inhibitory efficacy, these phytochemicals were prioritized for further characterization, including ADMET profiling and interaction studies, to evaluate their therapeutic potential.

**TABLE 1:** Docking outputs of the selected molecules. The table summarizes the docking results of the identified molecules, including their binding affinity, ligand efficiency, and interaction with CathB.

S. No.	Ligand ID	Phytochemical name	Affinity (kcal/mol)	Ligand efficiency (kcal/mol/non-H atom)
1	IMPHY001309	Anabsinthin	−9.4	0.2611
2	IMPHY010476	Withametelin F	−9.3	0.2818
3	IMPHY009003	Picrasidine M	−9.3	0.2514
4	IMPHY004234	Neochlorogenin	−9.2	0.2968
5	IMPHY011941	Paniculogenin	−9.2	0.2875
6	IMPHY010989	Nicandrenone	−9.2	0.2706
7	IMPHY007679	Bismurrayaquinone A	−9.1	0.2844
8	IMPHY011943	Neosolaspigenin	−9.1	0.2844
9	IMPHY007015	Solanocapsine	−9.0	0.2903
10	IMPHY014742	1β-hydroxycrabbogenin	−9.0	0.2903
11	Ca-074Me	N/A	−6.5	0.2321

### 3.2 ADMET analysis

The ADMET profiles of the top ten selected compounds were evaluated to assess their drug-like properties. Various ADMET parameters were analyzed, revealing similarities among the compounds. Notably, Nicandrenone and Picrasidine M stood out due to their superior ADMET characteristics, with no PAINS or toxicity profiles such as hERG blocker toxicity or hepatotoxicity (Table 2). Importantly, both selected compounds did not show any immunotoxicity or carcinogenicity in the computational toxicity predictions, indicating a lower probability of adverse immune responses or cancer-related risks upon administration. Both compounds demonstrated blood-brain barrier (BBB) permeability, making them potential candidates for treating AD and TBI. Nicandrenone and Picrasidine M exhibited high human intestinal absorption (HIA), moderate water solubility, acceptable BBB permeability, and no impact on CYP2D6 inhibition or OCT2 substrate activity. Therefore, both were selected for subsequent interaction analysis.

**TABLE 2:** ADMET profiles of the identified hit molecules. The table outlines the ADMET (absorption, distribution, metabolism, excretion, and toxicity) properties of the selected hit molecules.

S. No.	Phytochemical	Absorption		Distribution	Metabolism	Excretion	Toxicity	
		*HIA*	*Bioavailability*	*BBB permeation*	*CYP2D6* *Inhibitor*	*OCT2 substrate*	*hERG blocker*	*Hepatotoxicity*
1	Anabsinthin	Absorbed	Yes	No	No	No	Safe	Toxic
2	Withametelin F	Absorbed	Yes	Yes	No	Yes	Safe	Toxic
3	Picrasidine M	Absorbed	No	Yes	No	Yes	Safe	Safe
4	Neochlorogenin	Absorbed	Yes	No	No	No	Toxic	Toxic
5	Paniculogenin	Absorbed	Yes	No	No	No	Toxic	Toxic
6	Nicandrenone	Absorbed	Yes	Yes	No	Yes	Safe	Safe
7	Bismurrayaquinone A	Absorbed	No	No	No	No	Toxic	Safe
8	Neosolaspigenin	Absorbed	Yes	No	No	No	Toxic	Toxic
9	Solanocapsine	Absorbed	Yes	Yes	No	No	Toxic	Toxic
10	1β-hydroxycrabbogenin	Absorbed	Yes	Yes	No	No	Toxic	Toxic
11	Ca-074Me	Absorbed	Yes	No	No	No	Safe	Safe

### 3.3 Interactions analysis

**FIGURE 1:**
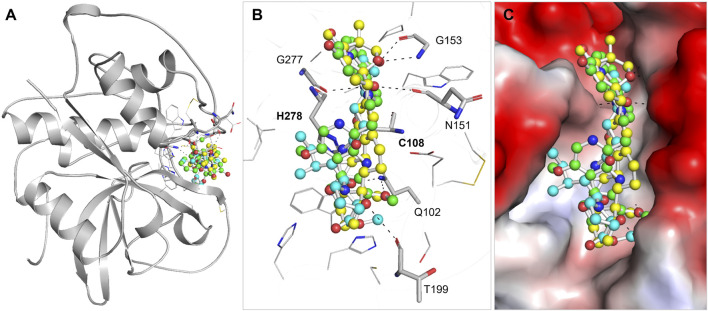
Structural depiction of (CathB) complexed with Nicandrenone, Picrasidine M, and Ca-074Me. **(A)** illustrates the cartoon representation of CathB in complex with the identified molecules Nicandrenone, Picrasidine M, and the control molecule Ca-074Me within the active site. **(B)** illustrates the magnified representation of the CathB binding pocket in complex with the identified molecules Nicandrenone (yellow), Picrasidine M (green), and the control molecule Ca-074Me (cyan) within the active site. **(C)** displays the corresponding charged surface views of the CathB binding pocket, showcasing the spatial occupation and interactions of the selected molecules within the enzyme’s active site.

PyMOL and LigPlus were used to analyze the interaction of Nicandrenone and Picrasidine M with CathB. Both compounds showed a similar binding pattern to the reference molecule, Ca-074Me (Figure 1). Ca-074Me was chosen as a reference inhibitor due to its potent and selective inhibition of CathB (IC_50_ = 2.24 nM) (Steverding, 2011). Its established role in CathB inhibition allows for a meaningful comparison with the identified phytochemicals. Nicandrenone and Picrasidine M formed multiple hydrogen bonds and hydrophobic interactions with key residues in the CathB binding site (Figure 1A). Notably, they interacted with critical residues such as Asn151, Gly153, Thr199, and Gly277 through hydrogen bonds and close hydrophobic interactions. The active site residues Cys108 and His278 were also directly involved in binding with both compounds (Figure 1B). These docking results demonstrated that Nicandrenone and Picrasidine M bind to the CathB binding pocket in a manner similar to the control molecule, Ca-074Me, within the deep binding pocket (Figure 1C). The findings suggest that both compounds can bind to the CathB active site and inhibit its catalytic activity, offering potential for therapeutic inhibition.

**FIGURE 2:**
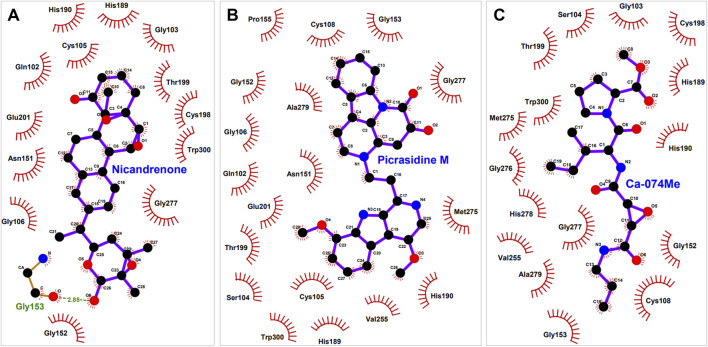
Interaction of (CathB) residues with selected molecules. This figure illustrates the key residues of CathB involved in interactions with **(A)**, Nicandrenone **(B)**, Picrasidine M and **(C)** the control molecule Ca-074Me. The interactions include hydrogen bonds, hydrophobic contacts, and other significant molecular interactions that contribute to the binding affinity of each molecule within the CathB active site.

Nicandrenone is a compound extracted from the leaves of *Nicandra physalodes*, a plant traditionally used for its various medicinal benefits. Nicandrenone has shown promise in studies for its ability to modulate certain biological pathways, contributing to its potential therapeutic applications. On the other hand, Picrasidine M is a bioactive alkaloid isolated from the root bark of *Picrasma quassioides*, a plant known for its medicinal properties. This compound has garnered attention for its potential pharmacological effects, including anti-inflammatory and anticancer activities (Mohd Jamil et al., 2020). It belongs to a class of alkaloids of interest for their therapeutic potential in various diseases. Both natural compounds demonstrate the significant pharmacological potential of plants in drug discovery. In the interaction study it was established that, such compound might inhibit CathB activity through shunning at the active sites. The binding modes of these compounds and the control molecule are shown in Figure 2, which contributes to the notion that they might be potential CathB inhibitors. Additionally, the quantitative analysis of the protein-ligand complexes was made through MD-simulations in order to compare the stability and alterations in these interactions.

### 3.4 MD simulation analysis

MD simulations are particularly essential for understanding the structure and function relationships and the stability of protein-ligand complexes (Shukla and Tripathi, 2020). In order to estimate the stability of CathB-ligand complexes and to determine the interactions of phytochemicals Nicandrenone and Picrasidine M with the CathB binding site, an all-atom MD simulation for 500 ns was performed. The trajectories were examined for various parameters such as RMSD (root mean square deviation), RMSF (root mean square fluctuation), *R*g (radius of gyration), and SASA (solvent accessible surface area), hydrogen bonding, and secondary structure as discussed in the subsequent sections.

#### 3.4.1 Structure dynamics

**FIGURE 3:**
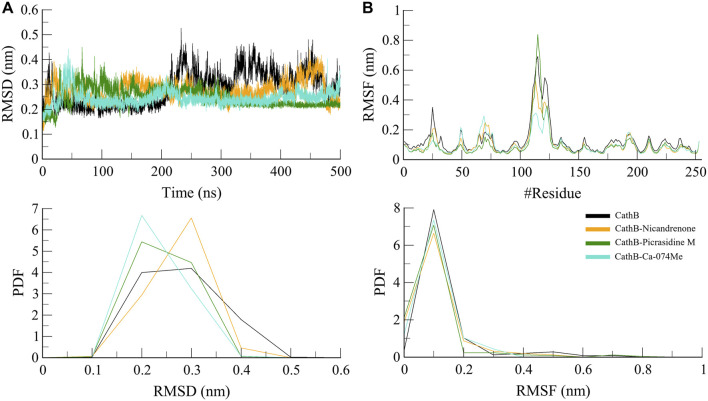
Structure dynamics analyses of CathB complexes during 500 ns molecular dynamics simulations. **(A)** presents the root mean square deviation (RMSD) plot, comparing the structural stability of CathB in its unbound state (black) and in complexes with Nicandrenone (orange), Picrasidine M (green), and Ca-074Me (cyan) over a 500 ns simulation period. **(B)** shows the root mean square fluctuation (RMSF) plot, highlighting the residual flexibility of CathB and its complexes with Nicandrenone, Picrasidine M, and Ca-074Me. The corresponding probability density function (PDF) graphs in the lower panels illustrate the distribution of RMSD and RMSF values, providing insights into the dynamic behavior of the protein and its complexes during the simulation.

The backbone RMSD function is applied as a measure to assess the general conformational stability of protein and the protein-ligand complex in the course of the MD simulations (Liu et al., 2017). A low RMSD value shows stability, thereby implying that there has been a little change in its structure, while a high RMSD value means that there has been a big change in conformation. In this study, RMSD trajectories revealed that the protein backbone in both free state and complex structure attained a plateau after the equilibration phase where the first 50 ns of the simulations were spend (Figure 3A). The free CathB protein had an average of 2.8 Å RMSD, while the CathB-Nicandrenone, CathB-Picrasidine M, and CathB-Ca-074Me complexes had average RMSD of 2.7 Å, 2.5 Å and 2.4 Å, respectively (Table 3). Consequently, these results imply that the binding of Nicandrenone, Picrasidine M, and Ca-074Me is involved in the stabilization of the CathB structure. However, the free protein had more fluctuations that suggest that the phytochemical binding to CathB provides stabilizing interactions of the complexes.

**TABLE 3:** The average molecular dynamics (MD) parameters for CathB and its complexes evaluated to assess their overall stability, flexibility, and structural characteristics during the simulation period.

Protein/Protein-ligand system	RMSD (nm)	RMSF (nm)	*R*g (nm)	SASA (nm^2^)	#H-bonds
CathB	0.28	0.13	1.8	128.3	159
CathB-Nicandrenone	0.27	0.10	1.8	126.7	162
CathB-Picrasidine M	0.25	0.10	1.8	128.0	160
CathB-Ca-074Me	0.24	0.09	1.8	128.2	166

Based on RMSF, the flexibility and residue-level variations of CathB were analyzed. This parameter calculates the mean distance of each amino acid residue from the starting position, providing information about a protein’s flexibility or rigidity. The RMSF analysis revealed that the free CathB protein had an average RMSF of 1.3 Å while the ligand-bound complexes CathB-Nicandrenone, CathB-Picrasidine M, and CathB-Ca-074Me had the lower average RMSF of 1.0 Å, 1.0 Å, 0.09 Å, as shown in Figure 3B. These observations indicate that the binding of the ligand limited the flexibility of the critical residues, especially those in the vicinity of, and within the binding site. The overall RMSF for all the systems fit into a similar pattern and any deviations that were noticed were minor suggesting that the structure was well conserved as the simulation proceeded. The active site residues were steady throughout the process of conformational changes, which indicates the stability of the protein-ligand binding.

#### 3.4.2 Structure compactness

**FIGURE 4:**
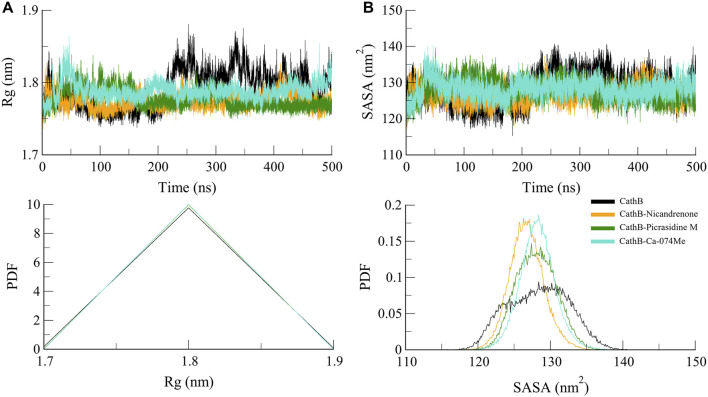
Structure compactness analyses of CathB complexes. **(A)** depicts the Radius of gyration (*R*g) plot, reflecting the compactness of CathB in its unbound state (black) and when bound to Nicandrenone (orange), Picrasidine M (green), and Ca-074Me (cyan) over the simulation period. **(B)** illustrates the solvent accessible surface area (SASA) plot, indicating that CathB exhibited higher solvent exposure in its unbound state compared to its complexes with Nicandrenone, Picrasidine M, and Ca-074Me. The lower panels show the probability distribution function (PDF) values for both *R*g and SASA, providing a comparative analysis of protein stability and solvent exposure among the complexes.

*R*g was calculated to determine the structural feature and folding propensity of CathB in free and ligand complex condition (Lobanov et al., 2008). This variable quantifies the atoms distance based on center of mass of protein it is useful in depicting conformational stability of the protein. The *R*g values of all systems were found to be nearly the same indicating that folding and overall contour structure of CathB was maintained consistently throughout the simulation study (Figure 4A). The *R*g values of all the ligand-bound complexes were less than by only a few nm that of the free protein, suggesting that the binding of Nicandrenone, Picrasidine M, and Ca-074Me did not bring about drastic alterations in the structure of the protein. This observation implies that the protein has not only compactness and stability structural characteristics but also losing them after the binding of the ligand. Moreover, the solvent exposure of CathB was examined by means of the SASA analysis. Specifically, the SASA refers to the part of the protein that is freely accessible to the solvent and hence gives information on folding and stability. The SASA plots were quite similar for the different trajectories, with the ligand-bound systems having slightly lower SASA values than the free protein (Figure 4B). This reduction proposes that the binding of Nicandrenone, Picrasidine M, and Ca-074Me lead to the change of conformation to a more compact one that is more stable and less exposed to solvent molecules.

#### 3.4.3 Hydrogen binding

**FIGURE 5:**
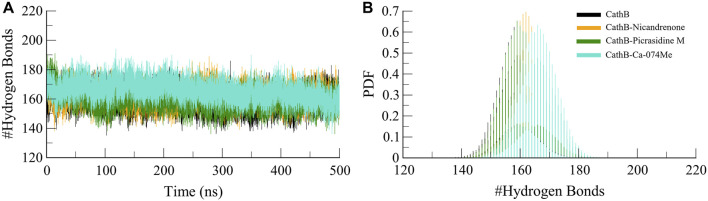
Analysis of intramolecular hydrogen bonds in CathB and its complexes. **(A)** displays the number of intramolecular hydrogen bonds within CathB in its unbound state (black) and in complexes with Nicandrenone (orange), Picrasidine M (green), and Ca-074Me (cyan) throughout the simulation period. **(B)** shows the probability distribution function (PDF) of intramolecular hydrogen bonds, highlighting the variations in hydrogen bonding patterns across the different states of CathB, indicative of structural stability and conformational changes.

Protein structures and protein-ligand complexes rely on hydrogen bonds as the main component that provides the stability and specificity of the complex (Williams and Ladbury, 2003). Molecular interactions between CathB and the bound ligands were studied using hydrogen bond analysis. The number and persistence of hydrogen bonds were obtained from MD trajectories (Figure 5). These simulation results indicated that most of the hydrogen bonds intramolecularly in CathB before and after ligand binding remained stable during the simulation, with distances varying between 2.5 and 3.5 Å (Figure 5A). Probability density function analysis of hydrogen bond distances also aligned with these results, suggesting a stable and recurring interaction over time (Figure 5B). Such stability of the hydrogen bonds shows that the CathB has strong and consistent binding with the phytochemicals without affecting its overall conformation.

**FIGURE 6:**
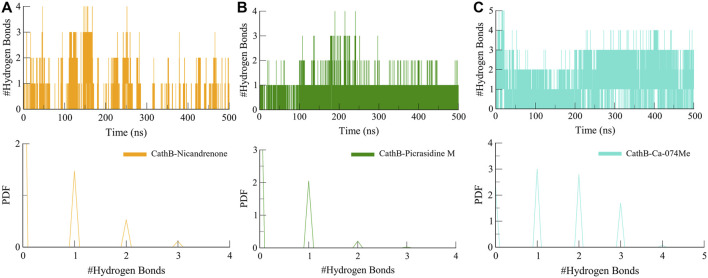
Dynamics of intermolecular hydrogen bonds. **(A)** Representation of intermolecular hydrogen bonds between CathB-Nicandrenone complex. **(B)** Representation of intermolecular hydrogen bonds between CathB-Picrasidine M complex. **(C)** Representation of intermolecular hydrogen bonds between CathB- Ca-074Me complex. The lower panels represent the distributed data points of RMSD and RMSF values.

Furthermore, intermolecular hydrogen bonds were analyzed to evaluate the stability and strength of the protein-ligand interactions within the CathB-Picrasidine M and CathB-Nicandrenone complexes. These bonds are crucial for maintaining the structural integrity of the complexes during dynamic simulations. The calculated number of intermolecular hydrogen bonds for the CathB-Nicandrenone and CathB-Picrasidine M complexes was up to four in each complex (Figure 6). The analysis revealed that both complexes maintained at least one intermolecular hydrogen bond throughout the simulation (Figures 6A, B). These bonds exhibited consistent distribution patterns and remained stable over the course of the simulation (Figure 6, lower panels). The persistent nature of these hydrogen bonds underscores the stability of the protein-ligand complexes, playing a key role in retaining ligand orientation and facilitating effective binding dynamics. These findings reinforce the structural robustness of the CathB complexes with Nicandrenone and Picrasidine M, highlighting their potential as promising candidates for further drug design studies.

#### 3.4.4 Secondary structure dynamics

**FIGURE 7:**
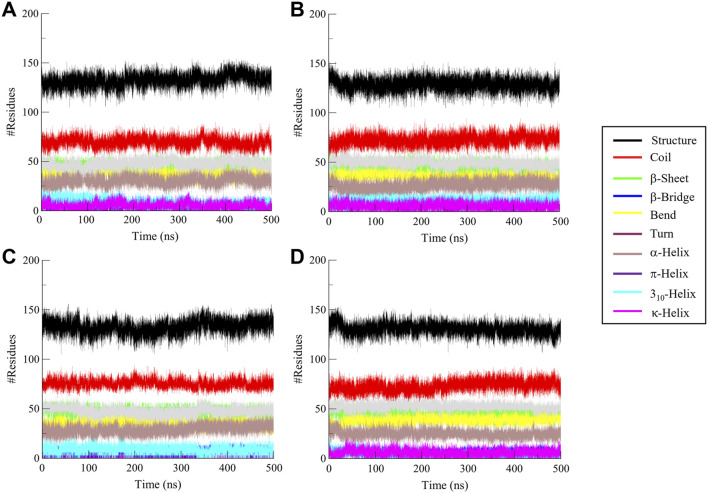
Secondary structure analysis of Cathepsin B (CathB) in complex with selected molecules. This figure illustrates the secondary structure elements of **(A)** CathB when bound to **(B)** Nicandrenone, **(C)** Picrasidine M, and **(D)** the control molecule Ca-074Me. Changes in α-helices, β-sheets, and other structural components are shown, providing insights into the structural integrity and conformational adaptations of CathB upon binding with the selected molecules.

The analysis of secondary structure dynamics provides insights into the structural stability and flexibility of proteins during simulations. By monitoring the transitions between different secondary structure elements, such as α-helices, β-sheets, and coils, we gain valuable insights into how the protein behaves under various conditions, including ligand binding. During the 500 ns simulation, the secondary structure elements such as α-helices, β-sheets, and coils were observed to check conformational transitions (Figure 7). The secondary structure analysis revealed that the secondary structure of CathB did not change in the free and ligand-bound states (Figures 7A–D). The ligand-bound systems preserved the secondary structural fragments present in the free protein, indicating that the binding of Nicandrenone, Picrasidine M, and Ca-074Me did not cause the protein to unfold (Table 4). The preservation of these structural fragments further supports the idea that the binding of CathB to these phytochemicals is stabilizing rather than disruptive. These findings are important as they suggest that the ligands are able to bind effectively without compromising the protein’s overall structural integrity, a key consideration in drug design for targeting CathB in therapeutic contexts.

**TABLE 4:** Average number of amino acids participating in secondary structure elements in CathB and its complexes. This table summarizes the average distribution of secondary structure components (α-helices, β-sheets, and coils) observed in the free and ligand-bound states of CathB during the 500 ns molecular dynamics simulation.

Secondary structure element	CathB	CathB-Nicandrenone	CathB-Picrasidine M	CathB-Ca-074Me
Coil	70	72	75	73
β-sheet	48	47	48	46
β-bridge	6	6	8	7
Bend	36	36	36	38
Turn	31	26	29	25
α-helix	48	49	48	53
π-helix	0	0	0	0
3_10_-helix	8	10	8	4
κ -Helix	5	6	0	6

### 3.5 Principal component analysis

**FIGURE 8:**
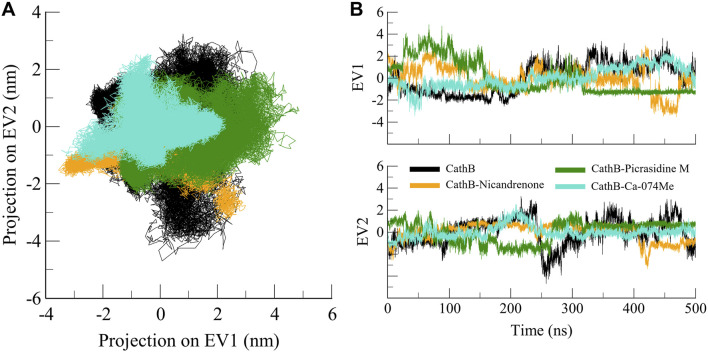
Conformational projection of CathB in principal component analysis. **(A)** 2D projection displays the results of PCA for CathB, CathB-Nicandrenone, CathB-Picrasidine M, and CathB-Ca-074Me. **(B)** Time-evolution trajectories of PCA for CathB, CathB-Nicandrenone, CathB-Picrasidine M, and CathB-Ca-074Me. Black, orange, green, and cyan represent CathB, CathB-Nicandrenone, CathB-Picrasidine M, and CathB-Ca-074Me, respectively.

PCA is a widely used approach to analyze the large collective movements of proteins and their complexes in the course of MD simulations (Moradi et al., 2024). It offers information about the conformational dynamics and stability of biomolecules by mapping the atomic trajectories onto principal components or eigenvectors. In this study, PCA was performed to compare the dynamic behavior of CathB in its free state and in complex with Nicandrenone, Picrasidine M, and Ca-074Me. The efficiency of conformational sampling of CathB and its complexes was analyzed based on the first two principal components of the covariance matrix of the *C*_α_ atoms motions. The outcomes, represented in Figure 8, show that the regions of conformational space available to CathB in the free form encompassed most of the conformations observed in the bound state. The black line shows free CathB, the orange line for CathB-Nicandrenone, green for CathB-Picrasidine M, and cyan for CathB-Ca-074Me systems. While some minor changes were noted for the complexes with Nicandrenone and Picrasidine M, these changes fell within the same class of conformation as the free-state (Figure 8A). The spatial patterns of movement reflected on the 2D map showed similar conformational changes in all four systems, with reduced conformational space occupancy following ligand binding (Figure 8B). The PCA analysis confirmed that the binding of Nicandrenone, Picrasidine M, and Ca-074Me did not significantly disrupt the dynamic motion of CathB, highlighting the stability and compatibility of these interactions. These findings underscore the robustness of CathB’s structural and dynamic properties, even upon ligand binding.

### 3.6 Free energy landscape analysis

**FIGURE 9:**
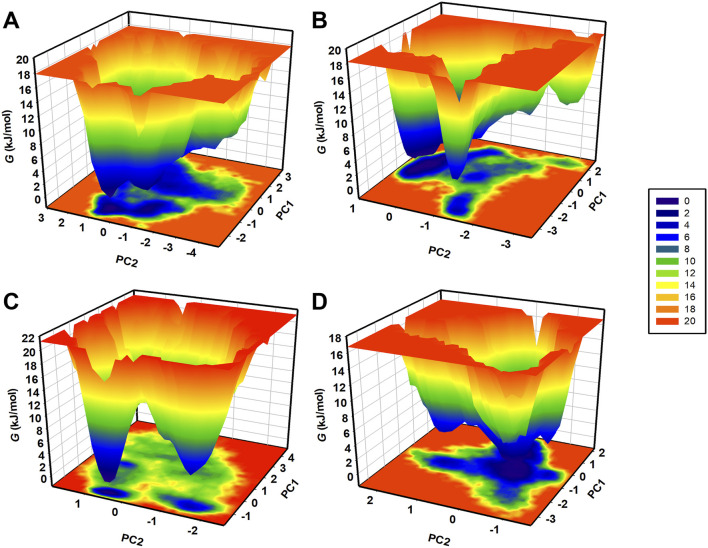
Free energy landscapes (FELs) for CathB **(A)** and its complexes **(B)** (CathB-Nicandrenone, **(C)** CathB-Picrasidine M, and **(D)** CathB-Ca-074Me) showing stable conformational states. Darker blue regions indicate low-energy conformations, while lighter regions represent higher-energy states.

The FEL is a valuable tool for representing the conformational stability and folding process in proteins and their complexes (Abdelsattar et al., 2021). MD trajectories were used to generate FELs based on the principal components (PCs) to address the energy minima and conformational states of CathB and its complexes with ligands. The FELs of CathB in its free form (Figure 9A) and when complexed with Nicandrenone, Picrasidine M, and Ca-074Me are shown in Figures 9B–D. In these plots, the lowest energy areas shaded in the darker blue correspond to stable conformations, whereas the areas of higher energy signify less favorable conformations. The CathB plot showed that the system possessed clear energy minima corresponding to conformations sampled during the simulations (Figure 9A). The binding of Nicandrenone and Picrasidine M with CathB only slightly influenced the size and position of the energy minima (Figures 9B, C). Nevertheless, these slight differences were insignificant, and the complexes retained one to two stable global minima as in FELs. This indicates that the binding of these ligands did not cause a large shift in the conformational stability of CathB. Comparisons of the FEL contours indicated that the ligand-bound systems had similar energy profiles and structural fluctuations as the free protein. These results, along with the PCA results, show that the binding of Nicandrenone, Picrasidine M, and Ca-074Me are thermodynamically favorable and do not disrupt the native fold of the protein (Figure 9D). The MD simulation trajectories and the analysis of the essential dynamics together provided support for the fact that Cathy and its complexes remained stable throughout the 500 ns simulation with slight changes in their conformations. The combination of PCA and FEL analysis offers a detailed picture of the dynamic behavior and conformational stability of CathB in the free and ligand-bound states.

### 3.7 MM/PBSA analysis

The MM/PBSA analysis was performed using the gmx_MMPBSA module in GROMACS to estimate the binding free energy of CathB protein-ligand complexes. This method provides an intrinsic thermodynamic evaluation of the energy variations accompanying ligand binding and relates the strength of the interaction to the stability of the bound system. The binding free energy components, namely, the van der Waals contributions and their standard deviations were determined and are detailed in Table 5. Results indicated strong binding affinities across all CathB-ligand complexes, suggesting stable interactions. Among these, the complex of CathB with Picrasidine M displayed the best binding affinity (−24.63 ± 3.00 kJ/mol), indicating tight binding, while the lowest binding free energy was found for CathB with Nicandrenone, indicating relatively less bindings stability. The entire screen revealed Picrasidine M and Nicandrenone as potent CathB binders, suggesting their therapeutic potential. Takentogether, while this study provides strong computational evidence supporting Nicandrenone and Picrasidine M as potential CathB inhibitors, experimental validation is necessary. Future work should include *in vitro* assays, *in vivo* pharmacokinetics, and selectivity studies to confirm their efficacy and therapeutic potential.

**TABLE 5:** MM-PBSA calculations of binding free energy for CathB-ligand complexes.

Complex	Δvdwaals	*ΔE*_*EL*_	Δ*E*_PB_	Δ*E*_NPOLAR_	Δ*G*_GAS_	Δ*G*_SOLV_	${∆G}_{Total\;\left(kJ/mol\right)}$
CathB-Nicandrenone	−8.03	−1.89	6.45	−1.01	−9.91	5.44	−4.47 ± 5.50
CathB-Picrasidine M	−46.65	−15.23	41.30	−4.05	−61.88	37.25	−24.63 ± 3.00
CathB-Ca-074Me	−33.65	−16.28	32.08	−3.45	−49.92	28.63	−21.29 ± 3.23

## 4 Conclusion

CathB has emerged as a promising therapeutic target in cancer, TBI, and AD because of its contributions to tumor invasion and neuronal loss. Here, in this study, a systematic virtual screening study was performed and identified two phytochemicals, Nicandrenone and Picrasidine M, with appreciable binding affinity, ligand efficiency, and selectivity for the CathB binding site compared with the control molecule Ca-074Me. Both phytochemicals showed promising pharmacokinetic properties and appropriate drug-likeliness with stabilized therapeutic profiles as anticancer and anti-inflammatory attributes. Interaction studies with the active site residues and all-atom simulations, along with MM/PBSA, PCA and FEL analyses, strengthened our understanding of the stability of these molecules in the CathB binding pocket. Taken together, Nicandrenone and Picrasidine M have a high potential to be used as promising leads for therapeutic development against various intricate diseases. However, more experimental works such as *in vitro* and *in vivo* analysis and clinical trials are needed to confirm their pharmacological potential. Additionally, selective inhibition of CathB over its isoforms remains a challenge, necessitating further structural modifications and validation studies to enhance specificity. Overall, the present study laid important groundwork for investigating new CathB inhibitors and their use in the treatment of cancer, TBI, and AD.

## Data Availability

The datasets presented in this study can be found in online repositories. The names of the repository/repositories and accession number(s) can be found in the article/supplementary material.
